# Bibliographical

**Published:** 1868-02

**Authors:** 


### BIBLIOGRAPHICAL.

Messrs. Sheldon & Connor, Book Sellers in this city, have laid on our table the following new publications, from the publishing houses of D. Appleton & Co., New York, and J. B. Lippincott & Co., Philadelphia.

*Physiology and Pathology of the Mind.* By Henry Maudsley, M. D., London Physician to the West London Hospital; Honorary Member of the Medico-Psycological Society of Paris; Formerly Resident Physician of the Manchester Royal Lunatic Hospital, etc.

We have delayed noticing this most able and instructive work, until we could get time to read it. We have done so, and do not hesitate to pronounce it a great work. To such as wish to study metaphysical and psycological science, there is no better work in the English language.

Every earnest enquirer into the mysterious subject of mental science, should read it.

To all of the learned professions, but especially the clergy, this book will be invaluable. The mechanical execution is good; four hnndred and fifty pages. D. Appleton & Co.

*Elements of Human Anatomy: General, Descriptive and Practical.* By J. G. Richardson, M. D., Professor of Anatomy in the Medical Department of the University of Louisiana. Second Edition, carefully revised, and illustrated by nearly three hundred engravings.

We could not say too much for this book, or its author. Dr. R. is a learned an earnest man, sincerely devoted to science, and pre-eminently qualified by his mental constitution for the task he has so thoroughly accomplished in the work before us. It is a work of six hundred and seventy pages. The mechanical execution is good. We commend it to the profession.

Dr. Samuel P. Hape, Surgeon Dentist, of this city, has laid upon our table the following new publications, from the publishing house of Lindsay & Blackiston, Philadelphia:

*The Practice of Medicine and Surgery. Applied to the Diseases and Accidents incident to Women.* By Wm. H. Byford, A. M., M. D., Author of a Treatise on the Chronic Inflammation and Displacement of the Unimpregnated Uterus, and Professor of Obstetrice and Diseases of Women and Children in the Chicago Medical College. Second edition; enlarged.

This is an excellent work, but on a subject upon which it is difficult to throw much new light; and yet the author has done this, and done it exceedingly well. We think this work equal to any now before the profession on the same subjects. Price $5; Lindsay & Backiston.

*Biennial Retrospect of Medicine and Surgery, and their Allied Sciences.* Edited by Mr. H. Power, Dr. Austin, Mr. Holmes, Mr. Thomas Windsor, Dr. Barnes, and Dr. C. Helton Fagge. For the New Sydenham Society. 500 pages, well bound.

This is a valuable book—an epitome of the present status of medicine and surgery and their allied sciences, and the literature of each. No physician should be without it. Lindsay & Blackiston, Philadelphia.

*Epidemic Meningitis; or Cerebro-Spinal Meningitis.* By Alfred Stille, M. D., Professor of Theory and Practice of

Medicine, and of Clinical Medicine, in the University of Pennsylvania; Physician to St. Joseph's Hospital, and to the Philadelphia Hospital. A monograph of 170 pages; neatly bound. Price $2.

This is the ablest work we have seen on this most interesting subject. We are pleased to note so large an amount of enquiry in this direction. Every inquiry of this kind brings us that far on our journey. The matrix of truth lies at the end of this road. The womb in which is concealed the true secret of morbid energy, the centre of etiological truth, where the weary, heart-sick pathologist may throw off his burden and rest from his laboos, will be found in the nervous centres, and in the ganglia of nerve cells. Lindsay & Blackiston.

*Head Aches; their Causes and their Cure.* By Henry G. Wright, M. D., M. R., C. S. L., L. S. A., Member of the Royal College of Physicians of England, Physician to the Samaritan Free Hospital, Fellow of the Royal Medical and Surgical Society, &c. From the Fourth London edition.

This is a most useful, excellent book. 150 pages. Lindsay & Blackiston. Price $1 25.

*Inhalation: Its Therapeutics and Practice. A Treatise on the inhalations of Gasses, Vapors, Nebulised Fluids and Powders, including a Description of the Apparatus employed, and a Record of Numerous Experiments, Physiological and Pathological, with Cases.* By J. Ellis Cohen, M. D. Illustrated.

This is an instructive pioneer work, full of suggestions upon a subject now attracting a large amount of attention. It has been gotten up in good taste. 200 pages. $2 50. Lindsay & Blackiston.

*Notes on the Origin, Nature, Prevention and Treatment of Asiatic Cholera.* By John C. Peters, M. D. Wih an Appendix.

This is an exhaustive monograph. Nothing more can be, said on this subject, from our present stand point. The author is undoubtedly a good observor and speaks ex cathedra.

After giving his own views of the pathology and treatment of cholera, he gives that of the Homœopathists also. We append what he says of them, and what they say of themselves:

"7.—HOMŒOPATHY AND CHOLERA."

"It is well known that the hydragogue cathartics, like elaterium, croton oil, jalap, gamboge, etc., are the truly homœopathic remedies for cholera. (See pages 140 and 165.) Yet, singularly enough, the homœopathists rarely or ever use them, but rely upon infinitesimal doses of more or less antagonistic and allopathic remedies, like camphor, copper, arsenic, etc.

The homœopathic treatment is generally commenced with camphor, which has been used from time immemorial against diarrhœa, ordinary cholera, etc. Leadam says that it was even used by Serapius, who translated Dioscorides into Syriac. But Hahnemann doubtless got the idea from much later sources, for he tells us in his Lesser writings (page 753) that "a receipt has been given to the world which has proved so efficacious against Asiatic cholera, that of ten patients but one died. The chief ingredient is camphor, which is in ten times the proportion ot the other ingredients." It is scarcely necessary to add that camphor has little or no homœpathic relation to cholera—certainly not as much as elaterium. Hahnemann and all his followers, also, instinctively avoid the use of infinitesimal or homœpathic doses of camphor, and the former directs strong spirits of camphor to be given at least every five minutes; also to rub some on the neck, head, arms, chest, abdomen, legs, etc.; also a clyster with two teaspoonfuls of spirits of camphor in one half pint of warm water; and, finally, that some camphor should be burned on a hot iron from time to time, so that the patient may inhale its vapors. This is very good treatment, but it is not homœopathic; on the contrary, camphor is an antidote to almost all homœopathic remedies and doses, which may be given subsequently. We have seen, on page 141, that veratrum, the remedy for the second stage, is not as successful as many homœopathists suppose, and it cannot well be in infinitesimal doses after the previous use of large doses of camphor, which antidotes it. The Hahnemannian remedy for the third stage, or that of cramps, when the patient is saturated, and his room and clothes loaded with the vapors of camphor, is one or two globules of the thirtieth dilution of cuprum, or copper. This is an allopathic astringent, but cannot act as such in infinitesimal doses. Even the use of copper was not original with Hahnemann, for Dupuytren and others had used it previously (see page 145,) and he tells us in his Lesser writings (page 755) that "trustworthy information from Hungary informs him that those who wore a plate of copper next the skin escaped the infection." We have shown, on pages 160 and 161, that few or none of the remedies in ordinary use by the homœpathists are homœpathic to cholera, as they do not use elaterium, etc., and it is almost safe to assume that they have never treated a case of cholera truly homœopathically. Hence, as they generally give infinitestimal doses of allopathic remedies, they must necessarily fail. They do not use their own remedies rightly, and a well-instructed regular physician can easily treat his cases, if he chooses, far more homœopathically than the oldest and most experienced homœopathist.

As it is not only easy, but natural to mistake various milder forms of disease for true Asiatic cholera, it is a matter of course that very many homœopathic physicians will rate their success very highly; others much more moderately. Thus two Cincinnati physicians say they treated one thousand one hundred and sixteen genuine cholera patients in 1849, with a loss of only thirty-five, or five and a half per cent.; Rubini, of Naples, five hundred and ninety-two cases, (with allopathic doses of camphor,) without a single death.

The *British Jaurnal of Homœopathy* (vol. 15, p. 130) says: 'Dr. Stens makes the rather rash assertion that the homœopathic mortality in cholera is only eight and a half per cent.' The British editor adds: "Now, we should rejoice very much were this the case; but, alas! we know from sad experience that it is at least three times as high as here stated. And this is a fact so easily ascertained by reference to the statistics of homœopathists themselves, that we (the British journalists) are surprised Dr. Stens has allowed such a flagrant exaggeration to damage the credibility of his other statements. We know very well the data on which the percentage of mortality he gives is founded, and we are well convinced of their utter untrustworthiness. How he could allow himself to put forward such an exaggeration, we are at a loss to imagine.'

The British journalists, of course, cannot believe Dr. Gerstel, who reported (see vol. 13, p. 329) to an Austrian Medical Society that he had treated three hundred cases of cholera, of a most inveterate character, with a loss of only thirty-two, or about ten per cent. An offer was made to Dr. Gerstel to practice under the control of the District Superintendent, Dr. Nushard, in order to establish proofs of the success of the homœpathic treatment, which he declined.

Dr. Rutherford Russell, one of the editors of the *British Journal of Homœpathy*, says, in vol. 7, p. 179:

"We cannot help deprecating the boastful tone we *so often* hear assumed by homœopathists on this subject—the treatment of cholera. It would argue a singular callousness of feeling in any one who has had much experience in the disease, at all events as it appeared among us, in Edinburgh, not to be penetrated with a profound sense of the comparative importance of our art in arresting, or even greatly modifying this terrible plague. In assuming what may be thought a tone of too great despondency as to the results of homœopathic treatment, we (Dr. Russell) refer to the fully developed disease. In its first stage, if we are permitted to see it at this time, much may be done to prevent its further development, and we cannot speak too strongly of the value of camphor; but in the stage of collapse, I have never seen any evidence of camphor being of service."

In the *British Journal of Homœpathy*, vol. 9, p. 693, we read: "We paid a visit to Dr. Tessier's hospital. He has one hundred beds; the wards are airy and high, and the hospital is well situated and served. He informed us that he had never met anything but uniform kindness and respect from the Central Bureau of Hospitals, although at various periods there have been medical men among them, and such is the case at present; not the slightest opposition has been offerec to him in the charge (from allopathic to homœopathic practice) that he has carried out in the medical treatment of his patients." This we know refers to the Hopital St. Marguerite, in which Tessier admits a loss of forty-eight or forty-nine per. cent. of his cholera cases. (See Hempel's and

Radde's Tessier on Cholera, p. 107.) The loss was only thirty-five to thirty-nine per cent. ot our quarantine last season; of six hundred and twenty-two cases of cholera and over fifteen hundred of diarrhœa, two hundred and forty-two died. Drs. Stens and Gerstel would doubtless have reported twenty-one hundred and twenty-two cases of cholera with about ten per cent. loss.

In vol. 12, p. 698, we learn that: "Dr. Tessier has been transferred from St. Marguerite to the Hospital Beaujon, one of the best regulated hospitals in Paris. His wards, male and female, contain one hundred beds. We are sorry to learn that the cholera has, in his wards, as well as in other hospitals in Paris, shown so malignant a type. One great cause for the increased mortality in all the hospitals, is the decidedly contagious character the disease has manifested. It thus spreads from bed to bed and attacks patients already suffering from serious diseases." I infer that the loss was still greater than in the Hospital St. Marguerite, and believe that Tessier has never publifhed any account of it.

In old times it might have been supposed and assumed that the contagiousness of the cholera in Tessier's wards accounted for the increased mortality. But we almost all believe in the contagiousness of cholera now, and cases occurring in a well appointed hospital, come earlier under treatment than under many other circumstances. The drawback that they have been or are sick with other diseases is somewhat counterbalanced by the facts that they do not have to be transported a great distance, are not half starved or racked with the pangs of exhaustion and debauchery, as many other cholera patients are, and that physicians, trained nurses, medicines, food, and every aid and comfort, are on the spot, for instant service, by night and day.

Besides, this loss from contagion occurred in 1854, when Dr. Budd, of Bristol, had established the great principles of disinfection (see page 100 of this treatise). Tessier, who is certainly an honest, earnest, and scientific homœopathist, neither knew how to prevent the infection, nor control after it had commenced.

Dr. Fleischman, of Vienna, has had the largest hospital experience of the homœopathic treatment of cholera (see *Brit. Jour. of Hom.*, vol. 14, p. 27); viz: twelve hundred and two cases, with seven hundred and ninety-three recoveries and four hundred and nine deaths. I know, from personal observation, that Fleischman's hospital is perfect in all its appointments. It is almost exquisite in its neatness, cleanliness, order. The consolations of religion are extended by the Sisters of Charity, and only the better class of the poor are admitted. The worst and most depraved classes find no entrance there. Yet Fleischman's results were only five per cent. better than on board the hospital-ship Falcon in our harbor last year. Fleischman candidly says: "In the treatment of this disease, at least, as we have it in a hospital, even for us homœopathists much remains to wish for. Every remedy which has been recommended has been tried and tried again by me, but I have little to say in praise of any of them."

Dr. Chargé, of Marseilles, received from the French Government the order of the Legion of Honor, and from Pope Pious IX. that of Gregory the Great, for services rendered in the cholera of 1849, in general practice. In the *British Journal of Homœopathy* (vol. 15, p. 173) we read: "In 1854 he was applied to by the Mayor of Marseilles to take charge of two cholera wards in the Hotel Dieu. All patients were to be sent on alternate days to the homœopathic and allopathic wards. It is true that Dr. Chargé resigned his trust after three reception days; it is also true that during those three days twenty-six patients were received and twenty-one died. Dr. Chargé complained that he had too few nurses allowed; that there was a great want of bed clothing, flannels, etc., that patients in other wards, when they took the cholera, as they often did, were transferred to the cholera wards; and, as this process of transfer was entirely in the hands of the allopathic medical officers, an opportunity was thereby afforded them of retaining in their own wards patients attacked by cholera on the day of the allopathic admission until the following day, when they might be thrust, in a dying state, into the homœopathic wards; and this, Dr. Chargè asserts, was frequently done."

This I cannot believe, but think the explanation is, that in 1849 Dr. Chargè was dealing with diarrhœa, cholera morbus, and cholerine, in private practice, and hence seemed very successful: while in 1854 he for the first time came in contact with true cholera, as it appears in general hospitals. Dr. Chargè certainly is not as able nor as scientific a physician as Tessier, and his success, we have seen, was not great, without any such imaginary unfair play.

Finally, Dr. Drysdale, one of the editors of the *British Journal of Homœopathy*, gives us, in vol. 8, some data by which we can form a prognosis under homœopathic treatment. He treated one hundred and seventy-five cases, of which forty-three or more were mild, with forty-five deaths. About tweuty cases seen in the first stage could not be saved. The cure of real choleraic, rice-water, painless diarrhœa, he says, was by no means an easy matter. Of those with severe cramps, twenty-two out of forty-six died; with coma, ten out of fourteen; with agonizing pain from the region of the heart, through the back, all, nine in number; died; with red purging, four, or all died; of the severe cases, without cramps, eight out of fourteen; with grinding of the teeth, four out of eight; with greenish tint of complexion, four, or all died; when purging was followed by cramps before vomiting, six out of nine died; all, (only two) died which commenced with fainting. If the vomiting began before the purging, four out of eleven died; if the purging preceded the vomiting, only six out of twenty-six proved fatal; with delirim, only four out of eleven died; with vomiting in gushes, only four out of ten; with hiccup, only two out of twelve; with epigastric pain, six out of twenty-four; with abdominal pain, six out of thirty-six; with moderate cramps, three of nineteen; and all those which commenced with colic recovered.

In the *consecutive fever*, of nine with coma, six died; with delirim, only two out of eight; with slow pulse, four out of twelve; with quick pulse, two out of four; with suppressed urine, two out of five; with restlessness, six out of sixteen; wth vomiting, three out of thirteen; with purging, six out of thirteen; with grinding of the teeth, three out of six; with sighing respiration, six out of ten; with sleeplessness, all, four in number, recovered; with headache, four out of six.

It is evident from all that has gone before, that many cases recover under all kinds of treatment, and under no treatment; and that many die under all varieties of treatment."

